# Analysis of the Impact of Intraoperative Margin Assessment with Adjunctive Use of MarginProbe versus Standard of Care on Tissue Volume Removed

**DOI:** 10.1155/2012/868623

**Published:** 2012-12-26

**Authors:** Ronald J. Rivera, Dennis R. Holmes, Lorraine Tafra

**Affiliations:** ^1^Los Angeles Center for Women's Health and David Geffen School of Medicine, University of California, Los Angeles, CA 90095, USA; ^2^Los Angeles Center for Women's Health and Keck School of Medicine, University of Southern California, Los Angeles, CA 90015, USA; ^3^Anne Arundel Medical Center, Annapolis, MD 21401, USA

## Abstract

Breast conserving surgery has been accepted as the optimal local therapy for women with early breast cancer, emphasizing the necessity to balance oncologic goals with patient satisfaction and cosmetic outcomes. In the move to enhance a surgeon's ability to achieve histologically clear margins intraoperatively at the initial surgery, the MarginProbe (Dune Medical Devices, Caesarea, Israel) has emerged as an effective tool to accomplish that task. Based on previously reported success using the device, we assessed cosmesis and tissue resection volumes among participants in a randomized-controlled trial comparing the standard of care lumpectomy performed with and without the MarginProbe. The use of the MarginProbe device resulted in a 57% reduction in reexcision rates compared to the control group with a small increase in tissue volume removed at the primary lumpectomy. When total tissue volumes removed were analyzed, the device and control groups were still very similar after normalization to bra cup size. We concluded that the MarginProbe is an effective device to assist surgeons in determining margin status intraoperatively while allowing for better patient cosmetic outcomes due to the smaller volumes of tissue resected and the reduction in patient referrals for second surgeries due to positive margins.

## 1. Introduction

Since the acceptance of breast conserving surgery with radiotherapy as a standard of care for early stage breast cancer [1], the National Comprehensive Cancer Network guidelines for breast cancer treatment recommend the assessment of surgical margins to aide in local control of disease. Any margins deemed positive should be considered for additional surgery according to these guidelines since clear surgical margins have been shown to minimize the risk of local recurrence [2].

The optimal time to identify positive margins is at the time of the initial operation since it affords the opportunity to reexcise positive or questionable margins without subjecting the patient to a second operation. The effort to achieve clear surgical margins intraoperatively is aided by surgeon's judgment, specimen palpation, gross sectioning, imaging [3], wire localization, frozen section, and touch prep analysis [4], among other techniques. In spite of these efforts, up to 40% of women in the USA continue to undergo multiple operations due to initial failure to achieve clear margins.

To further reduce the need for reexcisions, the MarginProbe (Dune Medical Devices, Caesarea, Israel) was developed to provide real-time, intraoperative assessment of the presence of disease at the surgical margins. The MarginProbe is a handheld device that utilizes radiofrequency spectroscopy to detect electromagnetic changes in malignant tissue within 1 mm of the margin surface. A 21-center randomized, controlled trial (Pivotal Trial) was conducted to determine if the adjunctive use of the MarginProbe would enhance standard of care practices employed by surgeons to reduce the need for reexcision procedures. While the complete results of this study are still awaiting publication, the use of the MarginProbe has been validated in other studies as an effective way to assess margins intraoperatively because of its high sensitivity in identifying malignant tissue and high specificity in distinguishing between normal and malignant tissues [5, 6].

A central question in the Pivotal Trial was whether or not the use of the MarginProbe device would result in the resection of excessively wide margins, thereby producing an adverse effect on cosmesis. Herein, we present the analysis of the cosmetic impact of intraoperative margin assessment using the MarginProbe on the participants in the Pivotal Trial. 

## 2. Materials and Methods

**Figure 1:**
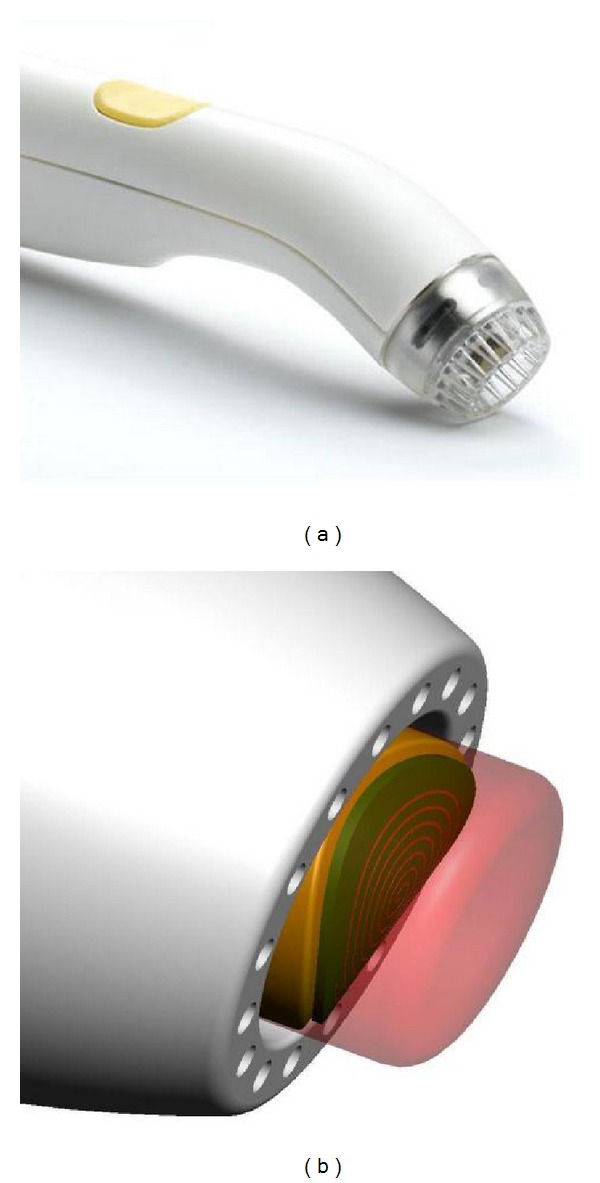
Photograph (a) of MarginProbe device showing vacuum mechanism that ensures full contact of the 7 mm sensor with the margin surface. Schematic (b) showing 7 mm diameter sensor and radiofrequency field that penetrates margin surface to detect cancer-associated electromagnetic changes.

**Figure 2:**
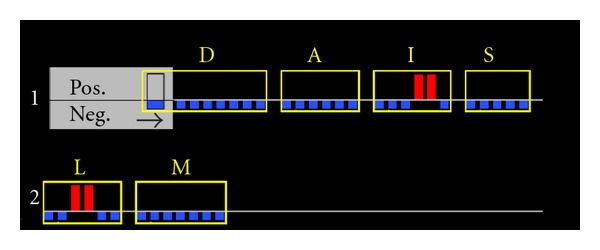
Visual binary signal display showing either positive (Pos.) or negative (Neg.) margin at multiple sites measured on each specimen margin surface [deep (D), anterior (A), inferior (I), superior (S), lateral (L), and medial (M)].

Six hundred and sixty-four (664) women with nonpalpable invasive cancer and/or DCIS undergoing lumpectomy were enrolled in the Pivotal Trial at 21 institutions. Following “standard of care (SOC)” lumpectomy, 596 women were randomized (1 : 1) intraoperatively to MarginProbe device use or control (i.e., SOC only with no device use). The definition of SOC varied by institution, but typically involved wire localization, specimen palpation, specimen radiography, and reexcision of questionable margins. Frozen section, touch prep analysis, and gross sectioning were not permitted to avoid confounding subsequent margin analysis. In women randomized to the device arm, the MarginProbe was used to assess each margin of the resected surgical specimen. The 7 mm sensor footplate at the probe tip was applied to a minimum of 5 sites and a maximum of 8 sites on each margin surface, depending on the area of each margin surface. A vacuum mechanism ensured full contact of the 7 mm sensor with the margin surface (Figure 1). An auditory and visual binary signal (positive/negative) was produced when the MarginProbe detected the presence or absence of malignant tissues within 1 mm of the margin surface at any of the 5–8 examined sites (Figure 2). By protocol requirements, any margin producing a positive reading required the reexcision of the entire affected margin. The thickness of each margin was left to the discretion of the operating surgeon. Skin margins and muscle margins did not require reexcision. Women randomized to the SOC arm underwent no additional margin resection following randomization. Surgeons were discouraged from taking additional shave margins as a safeguard against randomization to the SOC arm. Excision of shave margins following randomization to the SOC arm was considered a protocol violation and resulted in censuring of the data.

All primary and reexcision specimens in both arms were submitted for standard histopathological examination by pathologists who were blinded to the study arm. Device readings were compared per specimen for histological assessment of the initially excised lumpectomy specimens. True positive device readings occurred when invasive breast cancer or DCIS was detected histologically less than 1 mm from original specimen margin. True negative device readings occurred when histopathology of the primary specimen revealed no malignant cells within 1 mm of the corresponding margin surface. 

The ability to correctly and intraoperatively identify *all *of the involved margins on the main specimen and reexcise them was defined as a correct Complete Surgical Resection (CSR). Correctness or incorrectness of CSR was defined based on permanent histology data. CSR was defined as correct only when *all *main specimen margins detected as positive by histology were reexcised intraoperatively.

## 3. Results

The breakdown of tissue volume removed is shown in Table 1. When analyzing the impact of the MarginProbe on reexcision rates, the use of the device resulted in a 57% reduction in reexcision compared to the control group (device: 42/298 (14.1%), Control: 98/298 (29.9%), 57% reduction, *P* < 0.0001). As a result of true positive and false positive device readings, there was a small increase in tissue volume removed at primary lumpectomy (15.6 cc and less than 2 shavings per patient). Among patients requiring reexcision of positive margins at a second operation, less tissue was ultimately removed in the device arm (device: 28.4 cc, control: 49.5 cc, a 43.4% reduction). When analyzing the total tissue volumes removed (all operations combined), resected tissue volume was only slightly greater (8.5 cc) in the device arm (2.6% greater when normalized to bra cup size). 

## 4. Discussion

It is well established that cosmesis after breast conserving surgery is affected by multiple variables. Among the most important are the need for reexcision as well as surgery the amount of tissue removed at the primary and secondary surgeries [7–9]. Accurate intraoperative assessment of surgical margins allows the tumor to be removed in one surgical procedure, thereby sparing patients the burden of a second breast operation. However, standard of care approaches for intraoperative margin assessment (e.g., palpation, gross-sectioning of the specimen, specimen imaging, wire localization, and frozen section or touch prep analysis) continues to be commonly associated with margin reexcision rates of 20–40%. Reexcision has been associated with the risk of postoperative infection, delays in the onset of adjuvant therapy, lower patient satisfaction, lower rates of cosmetic acceptability, increased medical costs, and stress for patients who sometimes needlessly elect mastectomy rather than risk another positive margin [7–11]. Based on the results of the Pivotal Trial, breast reexcisions can be significantly reduced with the use of the MarginProbe device which should, in turn, significantly improve the safety and feasibility of breast conserving surgery. 

In spite of its benefits in reducing breast reexcisions, concerns have been expressed that the adjunctive use of the MarginProbe might compromise breast cosmesis due to the excessive resection of breast tissue, particularly when false positive readings are encountered. However, comparison of the device and control arms in the Pivotal Trial showed minimal impact on breast cosmesis when analyzed by the volume of resected breast tissue. In fact, only 2.6% greater volume was resected in the device arm when normalized to breast size. This corresponded to 2 additional margin shavings per patient, which is less than the 4–6 margins that may be indiscriminately reexcised by some surgeons who routinely harvest margin shavings. Furthermore, among patients who ultimately required reexcision at a second operation, there was essentially no difference in the two study arms (−1.6% difference, normalized) in the total volume of resected breast tissue. Collectively, these findings resolved concerns that the use of the MarginProbe may adversely affect cosmesis. 

## 5. Conclusion

The primary goal of breast conserving surgery is resection of breast malignancy with clear margins and acceptable cosmesis. While this goal is not always achievable at the initial operation, every reasonable effort should be made avoid multiple surgeries, undesirable cosmetic outcomes, increased treatment burden, and increased medical costs associated with these factors. The MarginProbe represents a practical advancement in the field of surgical specimen margin evaluation. When combined with the standard of care techniques, the MarginProbe may significantly lower the rates of reexcision for breast cancer patients, achieve comparable tissue volume removal at the first surgery, and reduce the amount of tissue removed among patient requiring a second operation. The end result is significant quality improvement in the management of conservatively treated breast cancer patients.

## Figures and Tables

**Table 1:** Total tissue volume removed.

Average per patient	Control	Device	Difference
Initial surgery			
Main specimen	61.3 cc	59.7 cc	−1.6 cc
True positive shavings	2.7 cc	6.7 cc	4.0 cc
False positive shavings	7.7 cc	21.1 cc	13.4 cc

Total volume	71.9 cc	87.5 cc	15.6 cc

Reexcision surgeries			
Tissue volume	49.5 cc	28.4 cc	−21.1 cc
Normalized tissue volume (normalized to breast volume)	5.6%	4.0%	−1.6%

All surgeries			
Total tissue volume	84.8 cc	93.3 cc	8.5 cc
Normalized total tissue volume (normalized to breast volume)	12.5%	15.1%	2.6%
